# Intracerebroventricular administration of *N*-acetylaspartylglutamate (NAAG) peptidase inhibitors is analgesic in inflammatory pain

**DOI:** 10.1186/1744-8069-4-31

**Published:** 2008-08-01

**Authors:** Tatsuo Yamamoto, Alan Kozikowski, Jia Zhou, Joseph H Neale

**Affiliations:** 1 Department of Anaesthesiology, Graduate School of Medicine, Chiba University, 1-8-1 Inohana, Chuo-ku, Chiba-shi, Chiba 260-8670, Japan; 2 Drug Discovery Program, Department of Medicinal Chemistry and Pharmacognosy, University of Illinois at Chicago, Chicago, Illinois 60612, USA; 3 PsychoGenics Inc., Tarrytown, NY 10591, USA; 4 Department of Biology, Georgetown University, Washington, D.C., 20057, USA; 5 Department of Anesthesiology at Kumamoto University, Kumamoto, Japan

## Abstract

**Background:**

The peptide neurotransmitter *N*-Acetylaspartylglutamate (NAAG) is the third most prevalent transmitter in the mammalian central nervous system. Local, intrathecal and systemic administration of inhibitors of enzymes that inactivate NAAG decrease responses to inflammatory pain in rat models. Consistent with NAAG's activation of group II metabotropic glutamate receptors, this analgesia is blocked by a group II antagonist.

**Results:**

This research aimed at determining if analgesia obtained following systemic administration of NAAG peptidase inhibitors is due to NAAG activation of group II mGluRs in brain circuits that mediate perception of inflammatory pain. NAAG and NAAG peptidase inhibitors, ZJ43 and 2-PMPA, were microinjected into a lateral ventricle prior to injection of formalin in the rat footpad. Each treatment reduced the early and late phases of the formalin-induced inflammatory pain response in a dose-dependent manner. The group II mGluR antagonist reversed these analgesic effects consistent with the conclusion that analgesia was mediated by increasing NAAG levels and the peptide's activation of group II receptors.

**Conclusion:**

These data contribute to proof of the concept that NAAG peptidase inhibition is a novel therapeutic approach to inflammatory pain and that these inhibitors achieve analgesia by elevating synaptic levels of NAAG within pain processing circuits in brain.

## Introduction

The peptide *N*-acetylaspartylglutamate (NAAG) is by far the most prevalent [1] and widely distributed co-transmitter in the mammalian nervous system[2,3]. It is co-expressed in discrete subsets of neurons with most small amine transmitters, including glutamate and GABA. Consistent with other neuropeptides, NAAG is released under conditions of high neuronal activity and acts at presynaptic receptors [4-6]. Synaptically released NAAG activates the group II metabotropic glutamate receptors [mGluR3 >> mGluR2; [6-8]]. These receptors are expressed on astrocytes where they stimulate release of trophic factors and on presynaptic axons where they inhibit transmitter release [5,6,9,10]. Two enzymes that inactivate synaptically released NAAG, glutamate carboxypeptidase II and III, have been cloned and characterized [11-15]. Potent inhibitors (IC_50 _= 1–5 nM) of these enzymes are being tested in animal models of neurological conditions that are mediated by high levels of glutamate release [16-18]. While these NAAG peptidase inhibitors do not possess direct agonist activity at ionotropic or metabotropic glutamate receptors, they, like group II mGluR agonists, are effective in reducing perception of inflammatory, neuropathic pain and bone cancer pain in rat models [19-24]. Consistent with the conclusion that inhibitors of NAAG peptidases achieve analgesia by elevating the degree of NAAG activation of a group II mGluR, group II antagonists completely reverse these analgesic actions.

While group II mGluR agonists influence nociceptive responses of primary sensory afferents [19,20,25-28], the widespread distribution of NAAG, NAAG peptidase activity [29] and group II mGluRs within pain pathways (reviewed in [30,31]) suggests that these receptors in the brain also might modulate pain perception following activation by NAAG. Group II mGluRs are upregulated in the central nervous system in response to inflammatory pain states [32-35]. In the periaquaductal grey, a brain region that contributes to descending modulation of nociceptive transmission within the spinal cord [36], group II mGluR agonists act presynaptically to reduce GABAergic transmission [37]. Speculation that this action contributes to analgesia derives from observations that opioid analgesia induced at the level of the periaquaductal grey also is mediated by reduction in GABAergic input to descending projections [38,39]. In this first test of the role of NAAG in regulation of pain perception via brain pain pathways, we administered NAAG and two NAAG peptidase inhibitors into the rat lateral ventricle prior to induction of inflammatory pain.

## Methods

These experiments were executed in adherence with the guidelines of the Committee for Research and Ethical Issues of the International Association for the Study of Pain (1983). They were performed according to a protocol approved by the Institutional Animal Care Committee of Chiba University, Chiba, Japan. Male Sprague-Dawley rats (250 – 300 g, Japan SLC, Shizuoka, Japan) were prepared with ICV catheters and examined for the effect of the agents on the formalin test of inflammatory pain.

### ICV cannulae

Implantation of the intracerebroventricular (ICV) injection cannula into the right lateral ventricle was performed stereotaxically under halothane anesthesia. Stainless steel guide cannulae (24 gauge, 0.64 mm outer diameter, 15 mm long) were stereotaxically placed through a burr hole (0.5 mm caudal to coronal suture and 1 mm lateral to sagital suture; 3 mm deep to the dura) and affixed to the skull with stainless steel screws and cranioplastic cement. In our experience, drug injection via the canulae is optimal about 4 days after implantation as the canulae have not plugged with cells by that time, in contrast to 7 days after implantation. Thus, ICV cannula implantation was performed 4 days before the formalin test. All animals displayed normal feeding and drinking behaviors postoperatively. Rats showing neurological deficits were not studied.

#### Formalin test

To carry out the formalin test, 50 μl of 5% formalin was injected subcutaneously (SC) into the dorsal surface of the right hind paw with a 25-gauge needle under brief halothane anesthesia. Within 1 min after the formalin injection, spontaneous flinching of the injected paw could be observed. Flinching is readily discriminated and is characterized as a rapid and brief withdrawal or flexion of the injected paw. This pain-related behavior was quantified by counting the number of flinches for 1 min periods at 1 – 2 and at 5 – 6 min, and then for 1 min periods at intervals during the period from 10 to 60 min after the injection. Two phases of spontaneous flinching behavior, an initial acute phase (phase 1: during the first 6 min after the formalin injection) and a prolonged tonic phase (phase 2: beginning about 10 min after the formalin injection), were observed. After the observation period, the animals were immediately killed with an overdose of barbiturate. Four to six rats were used for each treatment group reported here.

#### Behavioral analysis

The general behavior of each rat was carefully observed and tested. Motor functions were evaluated by the performance of two specific behavioral tasks, as follows. 1) The placing/stepping reflex: this response was evoked by drawing the dorsum of either hindpaw over the edge of a table top. In normal animals, this stimulus elicits an upward lifting of the paw onto the surface of the table, called stepping. Animals with any degree of hind limb flaccidity will demonstrate an altered or absent reflex. 2) The righting reflex: an animal placed horizontally with its back on the table will normally show an immediate coordinated twisting of the body around its longitudinal axis to regain its normal position on its feet. Animals displaying ataxic behavior will show a decreased ability to right themselves. To quantify the evaluation of motor functions, both tasks were scored on a scale of 0 to 2 in which 0 = absence of function and 2 = normal motor functions. Animals that were able to perform the motor tasks but did so more slowly than normal animals were assigned a score of 1. For example, the reflex withdrawal is typically immediate. Rats demonstrating a non-immediate reflex were scored 1. The normal righting reflex also is prompt and successful. Rats that either delayed the attempt or who were ultimately but not immediately successful were scored 1.

#### Drugs

(*S*)-2-[3-[(*S*)-1-carboxy-3-methylbutyl]ureido]pentanedioic acid (ZJ43) was synthesized following methods previously described [40]. ZJ43 (molecular weight = 304.3) is an unsymmetrical urea, which was prepared by the addition reaction of an isocyanate, generated in situ from the tosylate salt of glutamic acid dibenzyl ester and triphosgene in the presence of Et_3_N, with the second amino acid benzyl ester component. The subsequent debenzylation of the key intermediate by the catalytic hydrogenation affords ZJ43 in the final form. 2-(phosphonomethyl) pentanedioic acid (2-PMPA, molecular weight = 314) was purchased from Alexis Biochemicals, (San Diego, CA, USA). ZJ45 and 2-PMPA are NAAG peptidase inhibitors [16-18]. NAAG was purchased from Tocris (Bristol, UK). LY341495, a highly selective group II metabotropic glutamate receptor antagonist [41], was purchased from Tocris. The ICV administered drugs were delivered in a total volume of 3 μl.

#### Experimental protocol

ZJ43, 2-PMPA or NAAG were administered ICV 10 min before the formalin injection. To obtain control data, vehicle (saline) was injected ICV (n = 5). To verify that the analgesic effect of ICV administered drugs on the formalin test was mediated by the activation a group II mGluR, 1 mg/kg of LY341495 was administered intraperitoneally (i.p.) 10 min before the ICV injection of drugs. The effect of intraperitoneal (i.p.) administration of 1 mg/kg of LY341495 on the formalin test also was examined.

#### Statistical analyses

For the dose-response analysis, data from phase 1 (0 – 6 min) and phase 2 (10 – 60 min) observations were considered separately. The cumulative instances of formalin-evoked flinches during the phase 1 and phase 2 were calculated for each rat. To evaluate the dose-dependence, one-way analysis of variance (ANOVA) was used. Published data strongly suggest that underlying mechanisms that generate the phase 1 response is different from those that generate the phase 2 response. Thus, for the dose-response analysis, we considered phase 1 and phase 2 responses as independent samples. For multiple comparisons, Tukey's test was used. For comparison of the pain response in the peptidase inhibitor group versus the group that was treated with inhibitor and the group II antagonist, the unpaired t-test (two tailed) was used.

Whenever appropriate, results are expressed as mean ± SEM. Critical values that reached a p < 0.05 level of significance were considered significant.

## Results

### Behavioral analysis

ICV injection of 100 μg of ZJ43, caused restlessness in all animals and 20% of the rats receiving 100 μg of ZJ43 scored 1 (impairment of motor function) in the placing/stepping reflex and righting reflex. After ICV injection of 10 μg or less of NAAG, ZJ43 or 2-PMPA, all animals scored 2 (normal motor function) in the placing/stepping reflex and righting reflex tests. As a result, 10 μg was the highest dose of ZJ43 and 2-PMPA injected into the lateral ventricle in this study. After the i.p. administration of 1 mg/kg of LY341495, all animals scored 2 (normal motor function) in the placing/stepping reflex and righting reflex tests.

### Responses in formalin model of inflammatory pain

**Figure 1:**
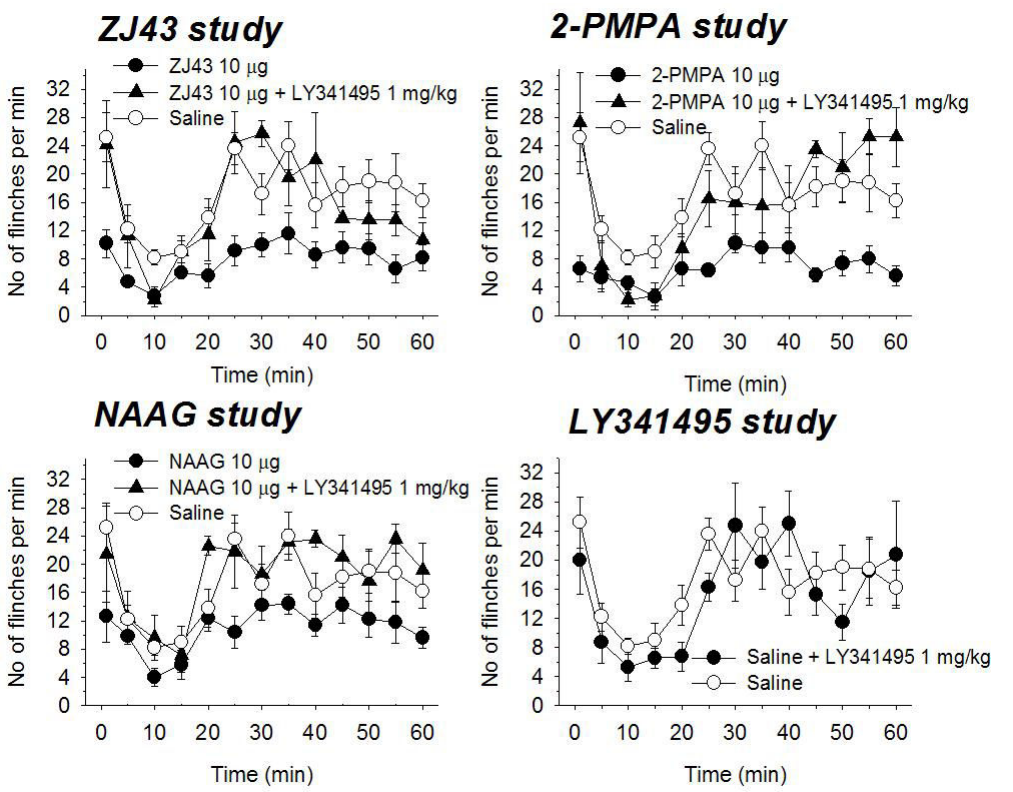
**Effects of intracerebroventricular (ICV) injection of 10 μg of ZJ43 (a), 2-PMPA (b) or NAAG (c) on the time course of the flinches observed after the formalin injection into the dorsal surface of the right rat hind-paw.** Drugs were administered 10 min before the formalin injection. The group II mGluR antagonist LY341495 (1mg/kg, i.p.) was injected 10 min before ZJ43, 2-PMPA, NAAG or saline (d). The number of flinches/min is plotted vs time after the formalin injection. NAAG and the NAAG peptidase inhibitors ZJ43 and 2-PMPA significantly reduced both phases of the response to formalin injection (see text for statistical assessment). The effects of the peptidase inhibitors were blocked by the LY341495 as was the effect of NAAG on phase 2 of the response to formalin. Each line represents the group mean and S.E.M. of four to six rats.

ICV injection of ZJ43 (Figure 1a), 2-PMPA (Figure 1b) or NAAG (Figure 1c) decreased the sum of flinches induced by formalin injection into the footpad while the group II mGluR agonist LY341495 alone (Figure 1c) had no detectable effect. Pretreatment with the group II mGluR antagonist LY341495 (1 mg/kg, i.p.) blocked the analgesic effect of 10 μg of ZJ43 or 2-PMPA on both phases of the flinching behavior (Figures 1a and 1b; phase 1: p < 0.01; phase 2: p < 0.01, by t-test). LY341495 similarly antagonized the analgesic effect of 10 μg of NAAG on the phase 2, but not phase 1, flinching behavior (Figure 1c; phase 1: p > 0.2; phase 2: p < 0.005, by *t*-test).

**Figure 2:**
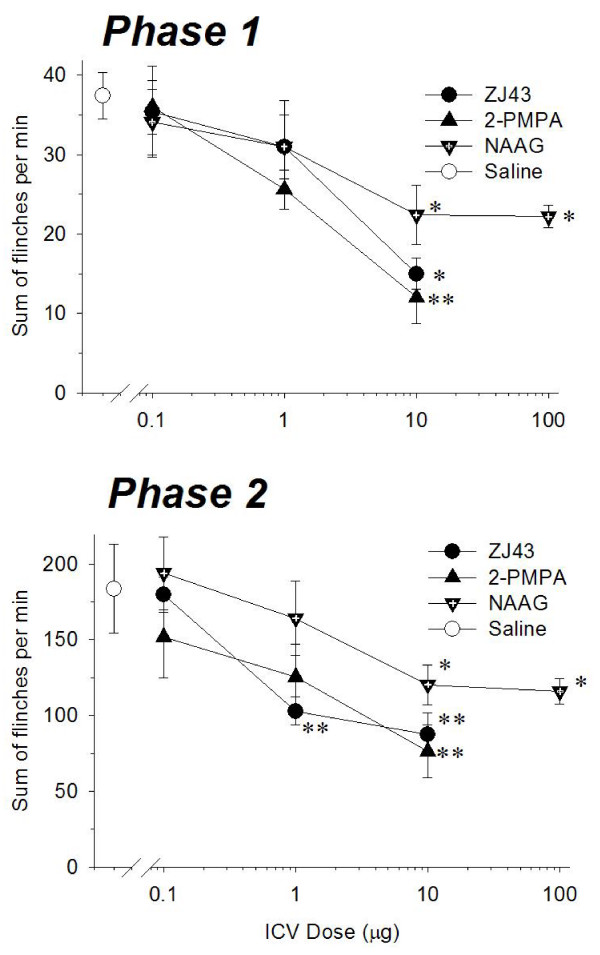
**Dose-response curves for ICV injection of ZJ43, 2-PMPA and NAAG representing the cumulative instances of formalin evoked flinches during the phase 1 (a) and the phase 2 (b).** ZJ43, 2-PMPA and NAAG reduced the number of phase 1 and the phase 2 flinching behaviors in a dose dependent manner. Each point represents the group mean and S.E.M. of responses by groups of 5–6 rats. * <0.05 and ** <0.005 versus rats given saline ICV prior to formalin-induced inflammation.

The NAAG peptidase inhibitors reduced phase 1 and phase 2 flinching behaviors in a dose-dependent manner relative to saline treated rats (Figures 2a and 2b; phase 1: p < 0.01; phase 2: p < 0.01 by ANOVA). Similarly, ICV injection of NAAG itself decreased the sum of flinches, in both phases of the flinching behavior in a dose-dependent manner between 1 and 10 μg (Figures 2a and 2b; phase 1: p < 0.05; phase 2: p < 0.01 by ANOVA). The maximal effect of the peptide appeared to be less than that obtained by the peptidase inhibitors. Ten μg of NAAG provided a maximal effect inasmuch as 100 μg of NAAG gave no greater reduction (Figure 2a and 2b).

## Discussion

We previously reported that systemic, intrathecal and local application of NAAG peptidase inhibitors reduced pain perception in rat models of inflammatory, neuropathic and bone cancer induced pain and that these effects are blocked by co-application of a group II mGluR [20-24]. These and other data support the conclusion that the analgesic effects of NAAG peptidase inhibition are mediated by increased activation of presynaptic group II mGluRs and a subsequent reduction in transmitter release in those neuronal circuits in which NAAG and the peptidase activity are expressed [reviewed in [16]]. However, it was not clear from these data if systemic application of NAAG peptidase inhibitors achieved analgesia by activation of group II mGluRs in brain pain pathways. This research ultimately is aimed at testing the concept that NAAG peptidase inhibition represents a clinically significant and completely new strategy for the treatment of inflammatory and neuropathic pain. As such, defining the locus of action of these peptidase inhibitors relative to pain perception is an issue of central importance. The data presented here support the conclusion that while the spinal cord and sensory neurons represent loci at which NAAG peptidase inhibition may contribute to analgesia, there also is at least one locus in the brain where NAAG directly mediates analgesia and that this brain region is accessible via the lateral ventricles. Given the quantities of the ZJ43 and 2-PMPA that are required locally [20] and intrathecally [21] to obtain analgesia, it is possible that the primary target for systemically applied NAAG peptidase inhibitor-mediated analgesia is in the brain and/or brain stem.

Since the inhibitors were infused into the right ventricle and the inflammation was induced in the ipsilateral footpad, it seems likely that the inhibitors were not acting proximal to this ventricle but rather that the compounds were affecting the broader periventricular tissue of the brain including the contralateral descending pain modulating pathway in the periaquaductal grey. The periaquaductal grey is an important locus in the pathway for opioid mediated analgesia. Relevant to these NAAG peptidase inhibition data, the periaquaductal grey contains a relatively high concentration of both NAAG and NAAG peptidase activity [29]. Further, group II mGluR agonist activation of presynaptic receptors on GABAergic neurons in the periaquaductal grey reduces GABA release [37]. We previously reported that NAAG inhibits GABA release from cortical neurons via a presynaptic mechanism [5]. A similar action of presynaptic opiate receptors on GABAergic neurons in the periaquaductal grey disinhibits a descending pathway that suppresses nociceptive transmission in the dorsal horn of the spinal cord [36,39]. While these data are consistent with speculation that intracerebroventricular administration of NAAG peptidase inhibitors might achieve analgesia via a mechanism that parallels that of the opiates in the PAG, a dose response study directly in the PAG is required to demonstrate this site of action. More importantly, these data together with our previous studies suggest that this peptide transmitter functions both spinally and centrally along pathways that mediate and modulate perception of inflammatory pain in a manner that is analogous to opiate peptides.

Given the wide distribution of group II mGluR receptors in the brain, it is surprising that direct infusion of NAAG did not result in a broad range of behavioral effects outside of the pain modulatory pathway and that its effects on the pain responses were not at least as profound as those of the peptidase inhibitors. This result is most likely due to the equally widespread distribution of extracellular NAAG peptidase activity in the brain. In the absence of peptidase inhibition, NAAG delivered by ICV is likely to be hydrolyzed rapidly as it diffuses through the extracellular space. Equally relevant to the development of new analgesic drugs, the systemic administration of the peptide would have the same drawbacks as synthetic group II agonists. That is, agonists activate receptors without a relationship to the normal level of information movement within circuits. In contrast, strategies that enhance the actions of endogenously released transmitters, such as NAAG peptidase inhibition, enhance the effects of the normal action of the circuit. This is, for example, the basis of the therapeutic efficacy of benzodiazepines, barbiturates and serotonin selective reuptake inhibitors.

ZJ43 is tricarboxylic acid with a urea core. Its hydrophilic structure contributes to its relatively low penetration across the CACO-2 cell culture model of tight junctions as found in the blood-brain barrier (Neale, et al., unpublished data). As a result, it is not surprising that despite its low nanomolar ^IC^50 for NAAG peptidases, relatively high (50–100 mg/kg) systemic doses of ZJ43 are required to obtain significant analgesia in inflammatory and neuropathic pain models [21]. This finding that some of the analgesic effects of ZJ43 are obtained by acting at pain circuits that are accessible via the lateral ventricle militates in favor of developing similarly potent NAAG peptidase inhibitors that cross the blood brain barrier more effectively. Toward this objective, we have synthesized a series of prodrug esters of ZJ43 that are themselves not NAAG peptidase inhibitors but that may enter the central nervous system more effectively due to their improved ClogP values. In preliminary studies, some of these prodrugs are more active in an inflammatory pain model than is ZJ43 (Yamamoto, unpublished). These results suggest that some esters of ZJ43 penetrate the blood-brain barrier effectively and are hydrolyzed efficiently by central nervous system esterases rather than by serum esterases. If NAAG peptidase inhibition continues to show promise as an analgesic pharmacotherapy, this prodrug development strategy is likely to be important in reducing the amount of drug required to achieve analgesia, thus reducing the probability of secondary effects and toxicity in other tissues.

## Conclusion

NAAG and its receptors are present in circuits within the brain and brainstem that process pain perception. This study provides the first direct demonstration that NAAG peptidase inhibitors reduce inflammatory pain perception by acting on pain communication pathways within the brain. Currently, clinical therapy for pain is limited to opioids and non-steroidal analgesics. This discovery of the analgesic effects of NAAG and NAAG peptidase inhibitors in the brain represents an important step in understanding the cellular mechanism underlying this promising new approach to analgesia.

## Abbreviations

N-acetylaspartylglutamate: NAAG; metabotropic glutamate receptor: mGluR.

## Competing interests

Tatsuo Yamamoto and Joseph Neale have no competing interests. This work was completed during an interval when Acenta Discovery held the license from Georgetown University to the intellectual property related to ZJ43. Dr. Kozikowski was the major owner of Acenta Discovery, Inc. Dr. Zhou was an employee of Acenta Discovery. Acenta Discovery is now owned by PyschoGenics, Inc., and Dr. Zhou is now an employee of PsychoGenics Inc. As such, Dr. Kozikowski and Dr. Zhou could profit from this class of compounds should they ever become drugs. Neither had any direct involvement in collection or analysis of data presented herein.

## Authors' contributions

TY executed the behavioral studies. JHN worked with AK and JZ on the characterization of ZJ43, generated the hypothesis, created the experimental design and wrote the manuscript. AK and JZ intellectual contribution was the designed ZJ43 within a series of related NAAG peptidase inhibitors. Additionally, JZ synthesized and chemically characterized ZJ43. All authors have reviewed and approved this final manuscript.
